# Quality of life in children after mild head injury

**Published:** 2008-08-15

**Authors:** Rotarescu Virginia, Ciurea A.V.

**Affiliations:** * Professor Chief of Neurosurgical Department Clinical Hospital “Bagdasar – Arseni”, “Carol Davila” University of Medicine and Pharmacy, Bucharest, Romania, MD, PhD; ** Senior Clinician Psychologist, Neuropsychological Laboratory of Clinical Hospital “Bagdasar – Arseni”, Postgraduate at “Carol Davila” University of Medicine and Pharmacy, Bucharest, Romania

**Keywords:** MHI, children, neurosurgical and neuropsychological evaluation, psychotherapy, QOL, reinsertion

## Abstract

**Introduction** The study was conducted to evaluate the effects of Mild Head Injury (MHI) in children not only in terms of impairment, but also in terms of disability, handicap and quality of life (QOL).

**Context:** Emergency Clinical Hospital “Bagdasar-Arseni”, Bucharest, Romania, between 2000 and 2004

**Methods** We take into account the patients with mild head injury MHI (CCS of 14 and 15 and amnesia). From a cohort of 1,319 children, consecutive patients with MHI, presented at the emergency room in a period of four years (2000-2003), 528 children (40.0%) were selected for admission, based on the presence of the risk factors. All admitted patients were investigated based on a protocol of neurosurgical evaluation and were followed for a period of 12 months.

**Results** The falls were the most common cause of MHI (30.6% - 162 cases). The proportion of children with detectable CT scan abnormalities was smaller (19.8% - 105 cases) and surgery was necessary in only 5.5% (29 cases). Special attention was paid to child-abuse and traffic accident cases. Post-concussion syndrome (PCS) was observed in 26.9% cases. Neuropsychological tests were performed in 96 children (21.2%), to evaluate neuropsychological, emotional, psychosocial and behavioral impairments. The study has shown that cognitive dysfunctions mainly were observed after MHI (especially deficits in information processing speed, memory and attention).

**Conclusions** The neurosurgeon should perform a complete evaluation of the children-patient with MHI, including a current physical examination, a neuro-radiological evaluation and a formal neuropsychological assessment, in order to detect the abnormalities and to treat them. Psychotherapy can be of benefit in cases with MHI. Any common case of MHI may hide a possible lesion with delayed consequences.

## Introduction

**Theoretical premises**

Traumatic brain injury (TBI) is the most frequent cause of neurological conditions in young people [**13**,**29**]. The most TBI are slight with loss of consciousness of five minutes or less, posttraumatic amnesia for less the 12 hours, and an initial score on Glasgow or Children Coma Scale of 13 to 15 [**40**]. Recent studies show that even slow TBI can determine neuropathological changes, leading to increasing number of rates for post-concussion syndrome (PCS) as a result of TBI [**11**,**28**]. The PCS symptoms are experienced of most patients with mild TBI [**17**, **18**, **23**, and **31**].

There are not specific criteria for PCS, although research criteria have been suggested [**4**]. Besides PCS, history, cognitive, somatic and affective changes are to be investigated [**3**, **6**, **8**, **9**, **10**, **12**, **19**, **20**, **24**, **25**, **26**, **27**, **30**, **32**, **33**, **35**, **36** and **37**]. 

Neuropsychological issues:

• Cognitive deficits can be seen even in patients without loss of consciousness and they affect the information processing speed, attention, memory and executive functions [**5**, **9**, **13**, **21**, **34**, **38**, **39**].

• The processing speed is diminished and the performance in multiple options-situations is disturbed [**14**, **17**, **43**].

• The distributive attention is more disturbed that the focused one or the sustained one (**35**, **37**, **38**, **43** and **45**).

• There can be some deficits for short-term memory, judgment, thinking, impulses control, organizable, planificatory and anticipatory behavior [**6**, **11**, **14**, **43**].

• The results in reproducing a complex geometrical form are poorer than in healthy subjects [**10**, **34**].

Specific neuro-psychiatrically issues:

• There is very difficult to make the difference between neurobiological and psychodynamic symptoms, and to the lack of the objective data (**1**, **8**, **11**).

• Malingering or seeking of secondary gain is often suspected in these patients (**16**, **22**).

• Doctors can be suspicious about patients with PCS and can accuse them for malingering or call them "neurotic", despite the presence of loss of attention, memory or executive functions (**2**, **41**, **42**, **44**, **46**).

• There is on over-estimation of secondary gain as a etiology of posttraumatic symptoms [**34**].

• Just a small percentage of patients eighth PCS have a poor and prolonged readjustment after a penal process or a potential compensation inquiry [**22**, **23**].

• PCS appears often in patients with good physical condition, without psychiatric history before the accident.

• The high frequency of PCS suggests a neurobiological origin exacerbated by psycho-dynamic factors (**15**, **24**, **33**, **40**).

Neurosurgical issues:

• This TBI represent a clinical-pathological heterogeneity which include the risk of the deterioration and for emergency surgery [**1**, **24**, **40**].

• The lack of therapeutic protocols and guides of leading to poor treatment for the patients and to resources' misuse.

• "The Head Injury Severity Scale"[**43**] is used to group this patients.

• According to this classification, patients with CCS from 14 to 15 plus PTA and loss of consciousness less than 5 minutes or disturbed memory and orientation are grouped in the diagnostic category MHI.

## Materials and Methods

The psychological research, to January 2000 from December 2004, designed as a longitudinal and transversal study on 96 children-patients presented in the Emergency Room and hospitalized with MHI. These were selected and distributed according to EPSEM principle (**E**qual **P**robability of **S**election **M**ethod), based on following criteria: CCS score between 14-15 p., age between 5 years and 16 years old and no associate conditions in order to eliminate the combined effects.

**Objective**

The longitudinal study is revealing the changes occurred in 12 months after MHI with individual visits at 3 months. At the study end, a quantitative processing of collected data prepared for significant correlations and conclusions. The final goal is to make comparison of these changes, the outcomes and the MHI consequences in children in order to evaluate the ability for reinsertion.

**Tools:**

Wechsler complete tests battery - WISC (Wechsler Intelligence Scale Children) – have ten specifics subtests, who describe different cognitive functions. We made this option because comparative itself product: before and after MHI.

Galveston Orientation and Amnesia Test (GOAT) emphasizes the space-time orientation and the presence of post-traumatic anterior-retrograde amnesia.

The "Rey Auditory Verbal Learning Test" emphasizes the volume of attention and memory according to age, work speed, the fatigability curve and an extensive range of behavioral and personality characteristics.

"Depression inventory for children”, developed by M.Kovacs, for the mood assessment.

The parents completed "The Social-Affective Scale" for children.

**Neuropsychological perspective**

**Sample description**

**Chart 1:**
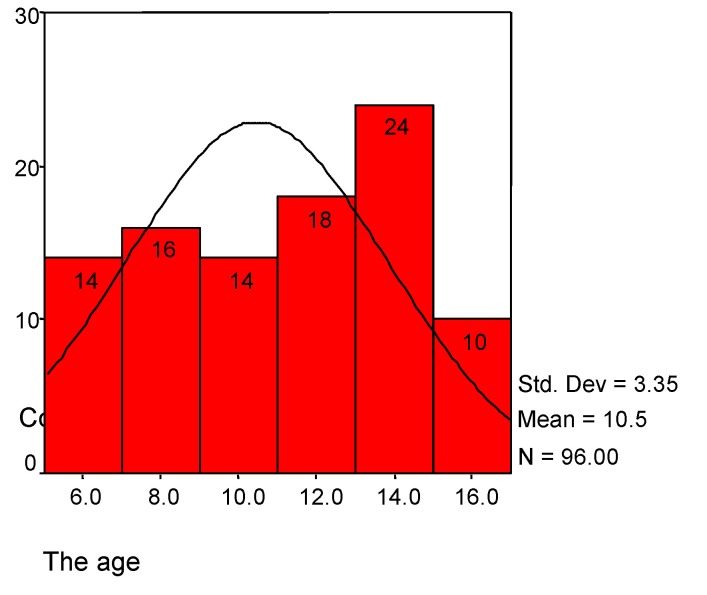
The age distribution in children

The mean of the age is 10.46 (SD=3.35); the distribution appeared like normal (See **Table 1** and **Charts 1**). The distribution by sex is preponderantly for male (70.8%).

**Table 1:** 

age		children	
Central tendency	N	Valid	96
		Missing	0
	Mean		10.46
	Median		11.00
	Std. Deviation		3. 35
	Minimum		5
	Maximum		15
Age groups	Years	Frequency	Percent
	5-7	22	22.9
	8-10	22	22.9
	11-13	26	27.1
	14-15	26	27.1
	Total	96	100.0
Sex distribution		Frequency	Percent
	Male	68	70.8
	Female	28	29.2
	Total	96	100.0

**Collecting data**

We established the first contact with the patients at 24 hours after admission, after medical check-up completed by the neurosurgeon (neurosurgical protocol), in order to offer data for a selection base. Initially, the GOAT was completed with all patients over 7 years (the normal score is between 76 and 100), in order to highlight the space-time orientation and amnesia. The mean score was 89.05 (SD=19.31) (see **Table 2**).

**Table 2:** GOAT

GOAT “Galveston Orientation and Amnesia Test” *	children	
	Frequency	Percent
Normal (76-100)	70	85.4
Borderline (65-75)	4	4.8
Deficit (<65)	8	9.8
N	Valid	82
	Missing	14
Mean		89.05
Minimum		0
Maximum		100
Standard deviation		19.31
* For Chi square=0.001, p<0.001		

We initially assessed the patients after the MHI in multiple sessions and they were following up and reassessed at 3, 6, 9 and 12 months. Structured interviews with the patients and their families were also completed for identifying the personal history and changes in the scholar and familial functioning. Our attention was focused on detection of hypothetic dissimulations, due to desire to obtain some secondary gains.

**Statistical analysis**

The collected data were descriptive and inferential processed with SPSS 10.05 (Statistical Package for Social Sciences). The first step was describing the distribution, main tendencies, variability and correlations. The second step, using the nonparametric inferential, was to estimate the probability of the aleatory deviations.

## Results

The neuropsychological recovery is not uniform among a group of patients, because each individual has his own recovery rhythm. Level of consciousness decreased immediately after the MHI, that is, a transient alteration of consciousness including partial or complete loss of consciousness. Amnesia includes the time preceding, during, and subsequent to the injury. The subjects presented preponderantly dizziness (41.7%) and Loss of Consciousness (LOC) for minutes (see **Table 3**).

**Table 3:** The consciousness state

Orientation in the time accident	Children	
	Frequency	Percent
Normal	2	2.1
Dizziness	40	41.7
Loss of consciousness for seconds	2	2.1
Loss of consciousness for minutes	38	39.6
Amnesia	14	14.6
N	96	100
* For Chi square=0.001, p<0.001		

In the next hours, the recorded deficits are not specific, but they concern efficiency information processing, execution and mental processes integration.

The causes of the injury were falls (60.4%), car accident (37.5%) and assault (2.1%). The demographic analysis shows: most patients are between 11-15 years old (mean age=10.5 years), people living in cities were more affected than people from villages were and boys more affected than girls.

**Cognitive outcomes**

**Attention**

**Chart 2:**
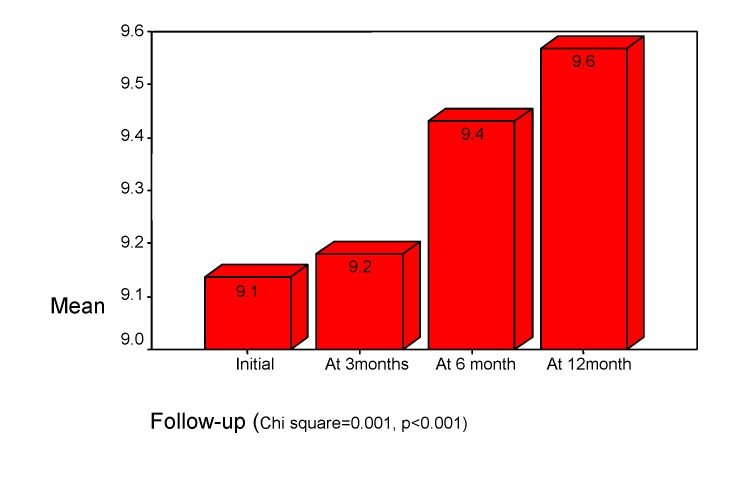
Focused attention. “Digit span" subtest of WISC

Focused attention and short-term memory were studied using the "digit span" subtest from WISC (normal standard score is 10). Initially, a slight deficit (mean score std. = 9.1) was identified in children (r=0.244, p<0.001), but this deficit is recovered in the future (See **Chart 2**). These deficits are obvious in developing an activity, in attention involvement in simple situations and in diminished the mental activity (Chi square=0.001, p<0.001).

**Chart 3:**
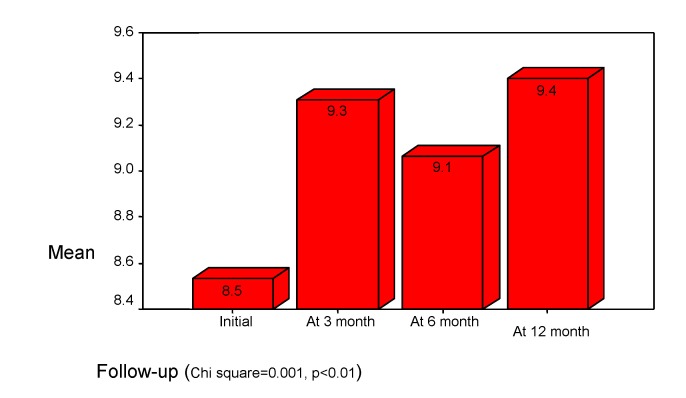
Divided Attention. "Digit symbol" subtest of WISC

The divided attention we studied with "digit symbol" subtest from WISC (the normal standard score is 10). The initial results show the mean score less than in focused attention (mean std. =8.5). The outcome is variable (see **Chart 3**).

**Chart 4:**
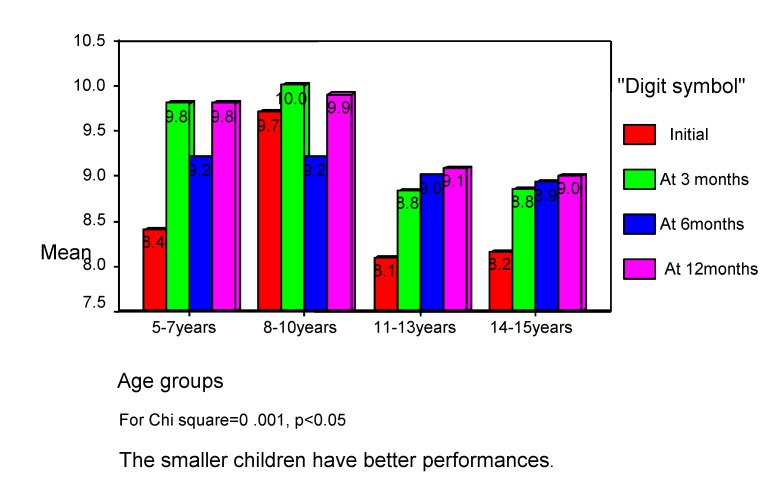
Divided attention correlate with age of children

Usually, the divided attention is more disturbed than focused attention, because there are some difficulties in maintaining and switching attention. The consequences are difficulties in performing efficient complex tasks, involving multiple simultaneous decisions, although the patients are capable to perform each simple task apart. The divided attention is more disturbed in the bigger age, so the smaller children have better performances (see **Chart 4**). 

**Chart 5:**
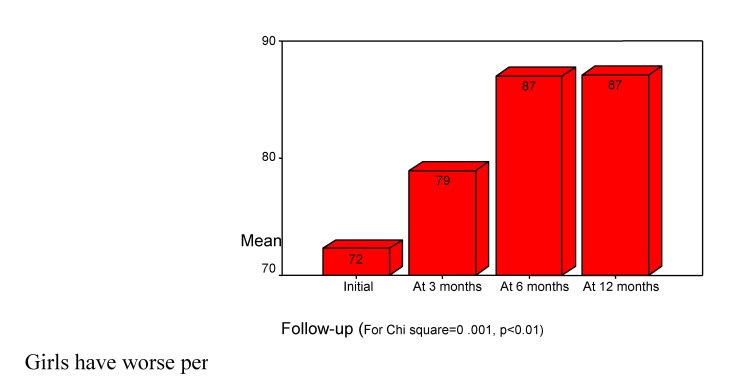
Children’s Volume of attention. "Rey Auditory Test" first trial

The attention volume we estimated using the results of first trial from “Auditory Verbal Learning Test” (Rey). The results were compared on a percentile scale, according to patient's age (the normal score is 100%; the slight deficit is flanked by 80%-99%; the medium deficit is between 40%-79% and severe deficit is less than 39%). Initially, children showed a decrease of attention volume (mean score 72%). (see **Chart 5**).

**Chart 6:**
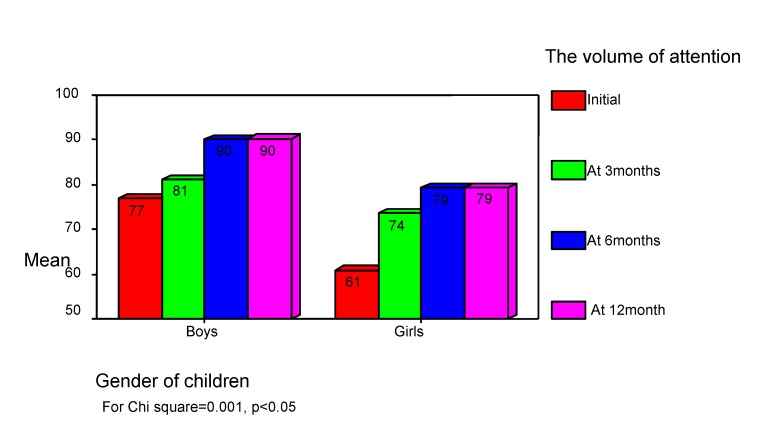
The volume of attention correlates with children gender. "Auditory Verbal Learning Test"

Girls have worse performances than boys do (see **Chart 6**).

**Chart 7:**
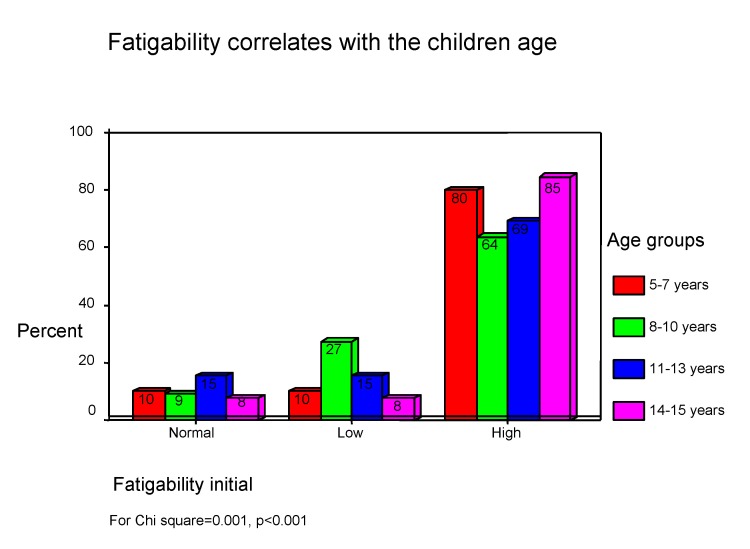
Initial fatigability in children

**Information processing capacity** is inferior because of deficits of adjustment and task integration (low attention volume) as a consequence of focusing efforts ("digit span"), difficulties in ocular-motor coordination ("digit symbols"), emotional distractibility and increased reactivity, low motivation and little interest along with increased tiredness. During the first few days after MHI, increased fatigability was present in children (See **Chart 7**) and it is positively associated with orientation and event remind capacity (GOAT) (Chi square=0.001, p<0.001).

**Chart 8:**
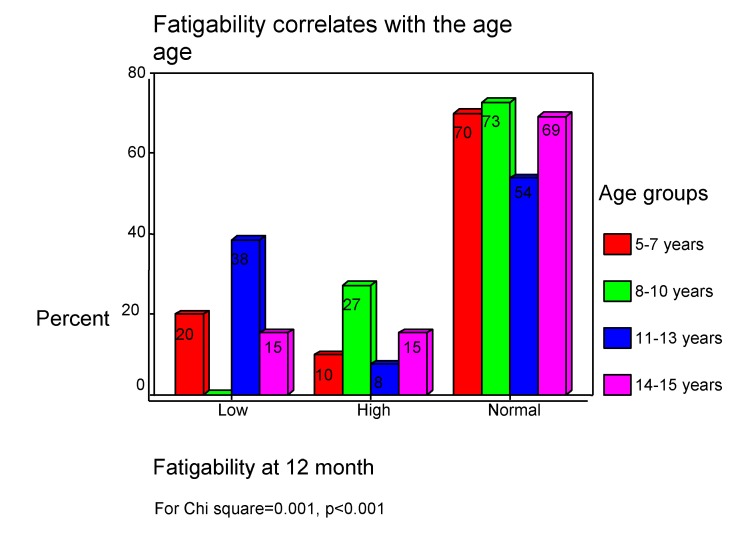
Fatigability in children after 12 months

After 12 months, in children there fatigability is absent (see **Chart 8**).

**Chart 9:**
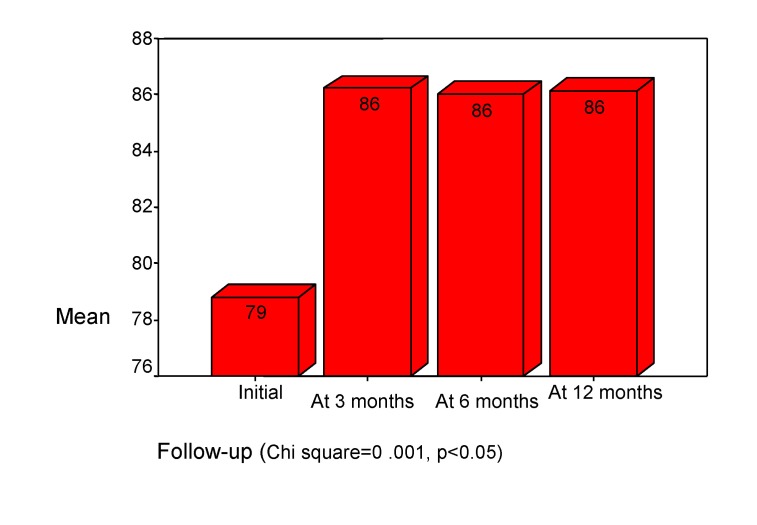
The volume of memory. “Auditory Verbal Learning Test" - 5th trial

Memory presented deficits for both: information access and information storage. These deficits were presented, interested in medium and long-term memory, for audible range. The memory volume we estimated with “Auditory Verbal Learning Test” and the results were compared on a percentile scale, according to the age (the normal volume score is 100%). The initial examination showed, in our study, a medium deficit of memory volume (mean= 79%). This increased after 3 months to 86% and was stabled in follow up. (See **Chart 9**) There is a negative correlation with age (rho= -0.283, p<0.001), with lower performances in older childhood (Chi square =0.001, p<0.05).

Visual memory we assessed with the "visual retention" subtest from Wechsler Memory Scale (to be watched three drawings, each for 10 sec. and than these drawings should be reproduced by the memory). The personal results are conversed directly in percentiles (the normal score is between 80%-100%). The mean standard score was 58% (for CCS=14p.) and 70% (for CCS=15p.). The age was in positive relationship with visual memory in children (rho=0.340, p<0.001), which suggests that extreme ages have visual memory with low performance. All these results lead to the conclusion that, in children, cognitive functions are disturbed by MHI because the brain functioning in global manner. Consecutively, the selection, the organization and the storage of information can be unsuccessful and the patient can ignore important details, which affect the accuracy of memory. The patients have difficulties in acquiring the new information and their habits so; in consequence, they are disturbed in daily routine.

**Integrative and conceptual thinking** we estimated with "objects assembly" subtest from WISC. There are some deficits in making spontaneous correlations. In our study, these deficits were presented in 22.7% of the children, with medium disability (13.6%) and severe disability (9.1%).

**Chart 10:**
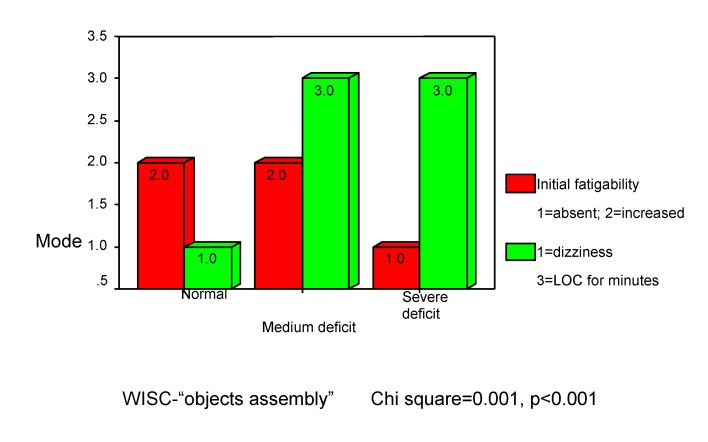
Integrative thinking

There are positive associations between "objects assembly" and “orientation at the accident's moment” (r =0.288, p<0.001), which suggests that “loss of consciousness for few minutes” (mode=3.0 inside **Chart 10**) leads to medium (“-“) or severe (“--“) deficits in terms of individual pathological significances (himself performance compare). There are negative associations between "objects assembly" and fatigability (rho=-0.420, p<0.001). In other words, severe deficit (--) is associated with low fatigability (mode=1.0 inside chart 10), which reinforce the relation between “deficit” and “loss of consciousness” (Chi square=0 .001, p<0.001).

The quality of conceptual thinking is altered.

The patient becomes unable to interpret correctly information: generalize or concretize. The verbal expression of his thoughts is not concise or accurate; the ideas can be imprecise in spoke manner with difficulties to find the appropriate words and the discourse can be dominated by pauses. Nonverbal interpretation beside a perceptive organization, with anticipations towards rapidly selection and a reason for decision, is deficiently and causes the decreased in flexibility and adaptation at unfamiliar.

**Premorbid characteristics**

**Structures of the sample**

**Chart 11:**
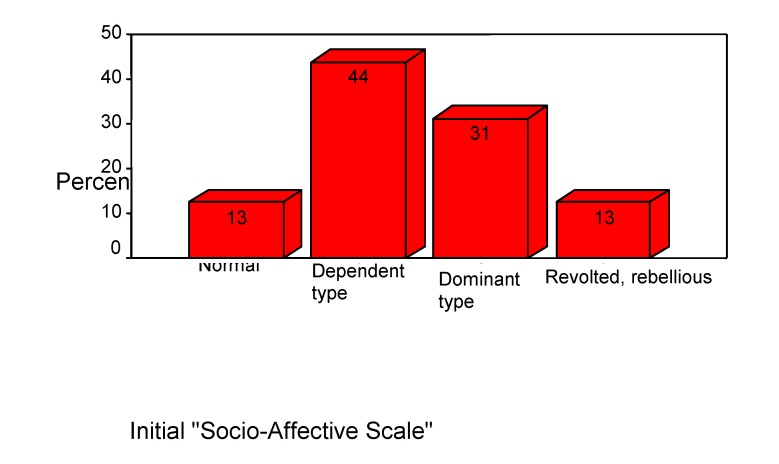
Premorbid characteristics in children

For children's premorbid assessment and to call attention to their outcome we used the "Social-Affective Scale". At the admission's time, 44% of children were dependent type, 31% dominant type, 13% were normal type and 12% rebel type. (See **Chart 11**)

**Mood and behavior changes**

**Chart 12:**
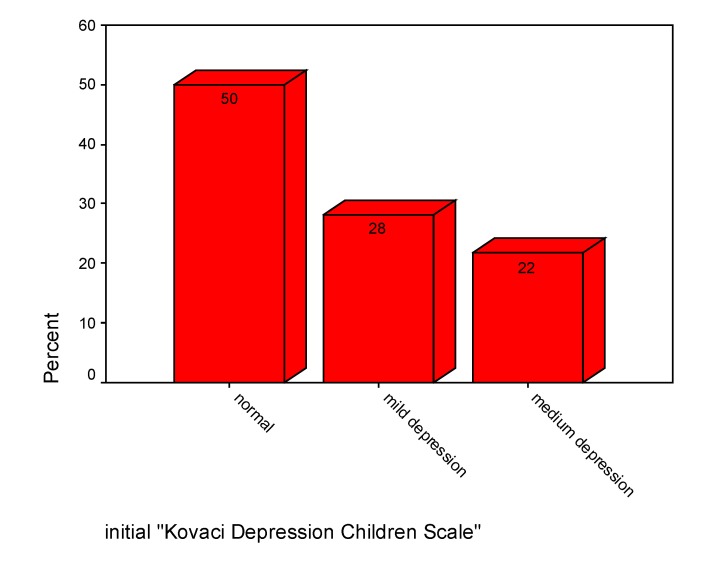
Initial mood

 "Kovacs children's depression rating scale" we used for assessed the mood. At admission, 50% of children were without depression, 28% had mild depression and 22% were with moderate depression (see **Chart 12**).

**Chart 13:**
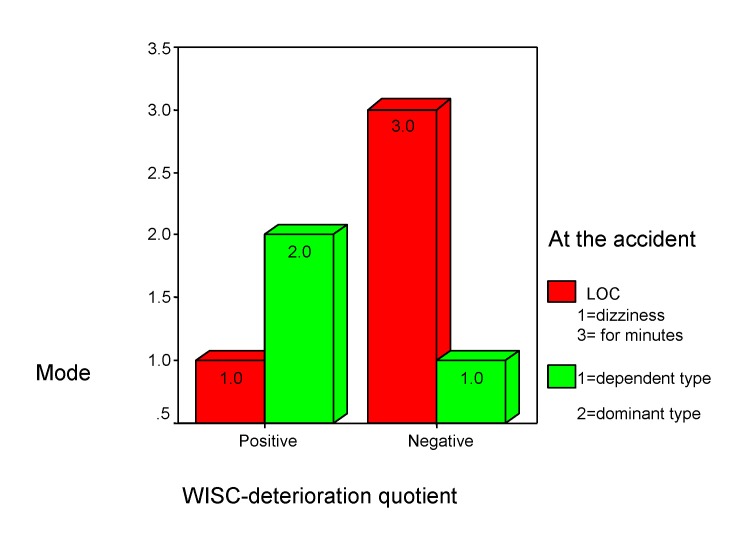
Children' predisposition

**Chart 14:**
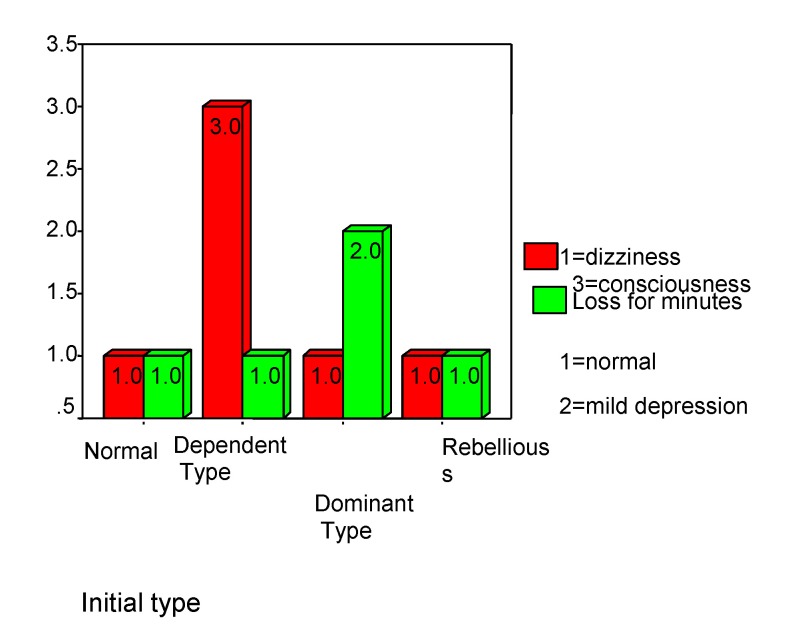
Children' predisposition

The dominant type is predisposed to loss by deterioration (rho=-0.388 p<0.001), even with a slight disturb of the consciousness as a consequences of the trauma (rho=0.353, p<0.001), while the dependent type is not affected by deterioration even if one suffers the loss of consciousness for few minutes (see **Chart 13**). A normal mood excludes loss of consciousness in MHI, and the “orientation at the time of the accident” is positively related to depression (rho=0.280, p<0.001) (see **Chart 14**).

**Chart 15:**
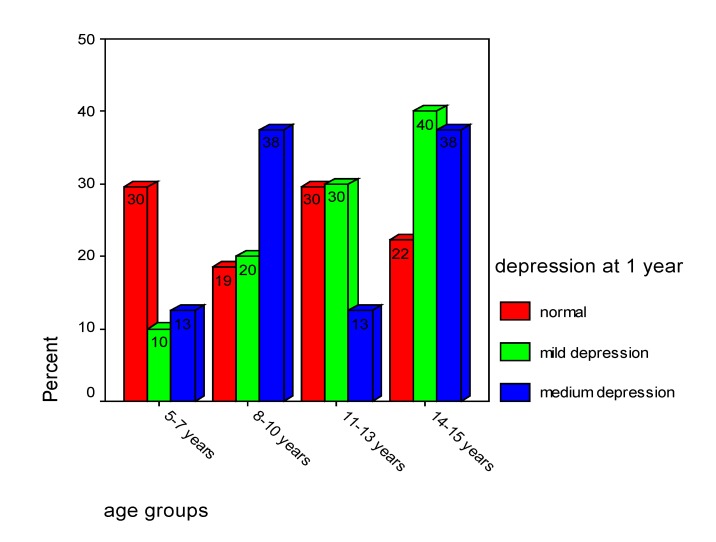
Depression

In our study, “loss of consciousness for seconds” is correlated with moderate depression for girls (within 14-15 years old) and with mild depression for boys (within 11-13 years old) (rho=0.376, p<0.001). At 12 months after MHI, medium depression was observed in 38% of the children inside 8-10 years old and 38% by individuals within 14-15 years old (**Chart 15**).

**Chart 16:**
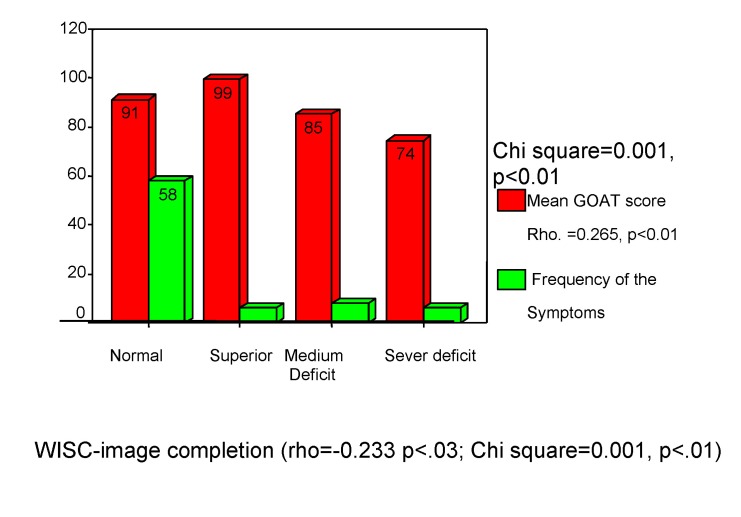
Symptoms and children’s performance

**Chart 17:**
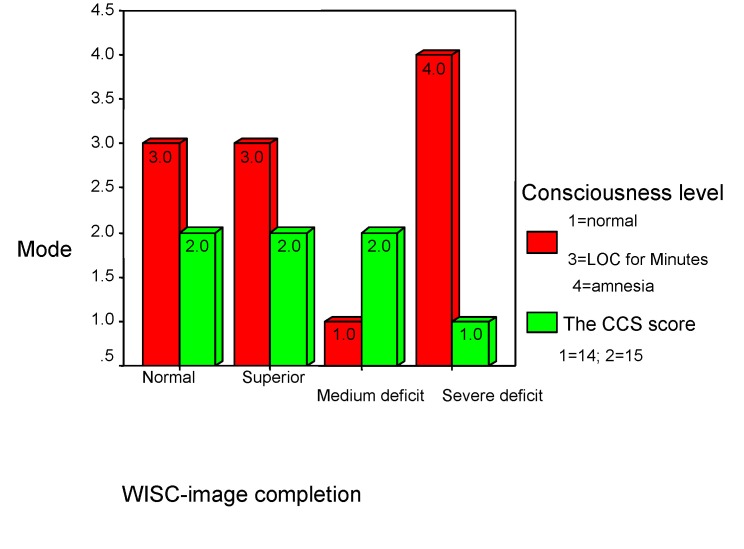
Consciousness level and children’s performance

**The symptoms** were correlated with WISC “Image completion” subtest (rho=-0.450, p<0.001) and with GOAT score (rho=0.265, p<0.001) in a dependence relationship. “Image completion” subtest results are influenced by the “orientation at the time of accident” (the symptoms depend of this), during hospitalization. There are not statistically significant correlations between the symptoms and premorbid characteristics. In evolution, the changes at these level are influenced by the CCS score (rho=0.276, p<0.001). The brain injury caused by the traumatism is suggested by these results; at a *14 points CCS score and with amnesia, it is recorded the severe deficit* at this test for 9% of the studied children. The normal framing of the results has been obtained for 75% of the patients, 7% scored superior and 9% scored a medium deficit (see **Charts 16**, **17**).

The symptoms were expressed or not, depending of endure requires, one year follow up after the MHI. The headache was most frequent, sometimes associated with tiredness or/and dizziness even after 12 months after suffering the accident. In our survey, four children (4.2%) described grand-mal crisis after 3 months and two children (2.1%) – after one year – were still describing them (see **Table 4**). There were not mixtures of stressfully events in the children’ social-familial environment during this study.

**Table 4:** Symptoms’ evolution in children

Symptoms	Initially	At 3 months	At 6 months	At 12 months
Absent		40%	50%	60%
1 persistent symptom	19%	27%	12,5%	10%
2 symptoms	13%	19%	17%	19%
3 or more symptoms	58%	14%	10,5%	13%

Most times, the symptoms are considerate as a new state to which they must adapt therefore spontaneous symptoms expression is frequently avoided in order not to be interpreted as wails, which could increase coping efforts.

Psychotherapy is an effective intervention and must be starting while the patient's hospitalization. The binomial comparison test of the obtained results through psychotherapy confirms the affirmations stated above and expresses the significant difference between the two groups.

**Table 5:** Binomial test

		Children			
Psychotherapy	Category	N	Observed Proportion	Test Proportion	Asymp. Sig. (1-tailed)
Group 1	No psychotherapy	44	.536585	.33	.000ª
Group 2	Yes psychotherapy	38	.46		
Total		82	1.00		
a Based on Z Approximation.					

Trough psychotherapy ascertain deficits are being corrected and it is obtained an improvement of personal performances which leads to a better adaptation on daily solicitations, thus improving life quality of the MHI affected persons. Good results were obtained for 74 % children with falls (for Chi square=0.044 and p<0.05).

**Chart 18:**
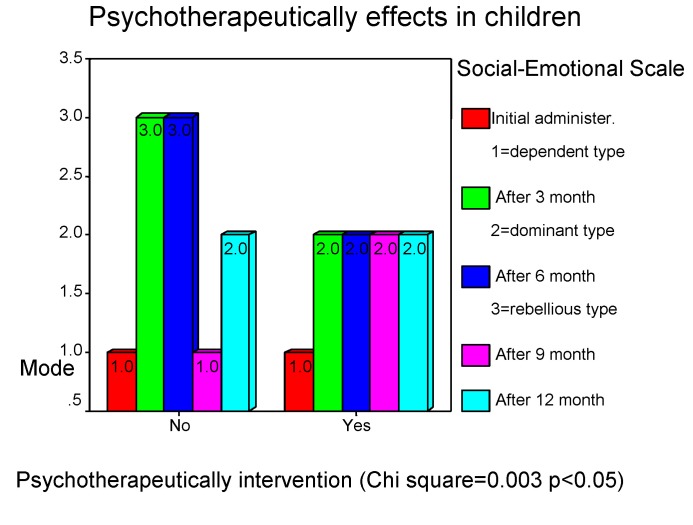
Psychotherapeutically effects in children

**Chart 19:**
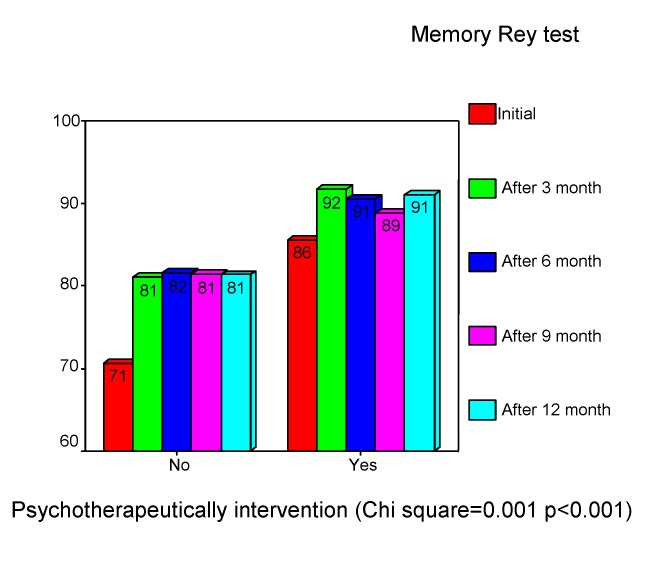
Psychotherapeutically effects in children

## Discussions and conclusions

The quality of life is an evaluative concept, resulted by comparing the conditions of life and the human life's activities with the human necessities, values and aspirations. It refers to the global evaluation of life (how good and satisfying is a person's or a group's life) as well as to the evaluation of the different conditions or spheres of life (familial, social, professional and interpersonal relationships' environment). The quality of life represents a different description to the concept of happiness (subjective state resulted from living one's own life) were the interest is focused mainly on determining the objective factors responsible for the life's quality variation and on the strategies of action in order to increase it.

Post injury behavior problems in patients with MHI may be associated with preinjury difficulties [**6**, **17**, **18**, **30**]. The following behaviors can be associated with MHI: hyperactivity, sleep disturbances, appetite problems, disturbances of motivation and social withdrawal [**31**, **33**, **34**]. Not all these deficits are obvious in common interactions, or in usual examinations. Most commonly, dysfunction is found in the patient’s social interactions, particularly in relation to recreation, work, school, and home [**5**, **13**, **33**, **37**, **38**]. Adapted neuropsychological examinations are necessary in order to discover them. Commonly, the patients complain of tiredness or reduction of their efficiency. Generally, these symptoms do not persist for a long period. They are only present for several weeks or months post injury and disappear gradually after 6 to 12 months. Usually, individuals with minor cognitive dysfunction can easily reintegrate their social and professional former condition. Nevertheless, sometimes emotional and behavioral problems may appear. There are some risk factors such as stress, fatigue and anxiety [**10**, **19**, **45**]. Persisting problems following MHI are more common in those with previous head injury, pre-existing learning difficulties, or neurological, psychiatric, or family problems. In our study, we excluded these by the last selection criteria (*no associate conditions in order to eliminate the combined effects*).

MHI continues to be a controversial matter regarding neurological sequelae [**7**, **8**, **20**, **27**] or psychological sequelae [**34**] after minor trauma. The existent data show that patients with more severe traumas express less emotional symptoms than patients with MHI [**27**]; the hypothesis is that more severe traumas disturb the ability of processing the self-consciousness. Memory deficits together with poor self-consciousness lead to easy forgetfulness of any stress agent. On the other hand, patients with MHI can have an increased self-consciousness of their own differences between how they were before and the manner they are after the trauma (e.g. decreased cognitive abilities or low speed in information's processing), and increased difficulties in emotional control [**37**, **38**].

The clinical picture is dominated by attention and memory deficits, disfunctioning praxis, perceptual-motor abilities (especially processing speed) and changes in patient's personality and temperament. All of this induce adaptation difficulties to daily schedule (social-educational) and lower the quality of life through the disagreeable emotional and affective feelings. Certain persons can perceive changes as repeated and unexpected failures; they will blame themselves and will probably develop a tendency to depression and anxiety. Their reduced capacity of solving the problems makes them impulsive and emotionally unstable [**5**, **6**, **10**, **13**, **19**]. The increase of anxiety conducts to decrease of cognitive performances by augmentation of disabilities. The anterior-retrograde amnesia and the synergetic interaction between cognitive deficits and anxiety incoming afterwards discern the MHI syndrome (not including nightmare) from the traditional posttraumatic stress syndrome (associated with nightmare, especially). Panic is the symptom that can mediate and increase the cognitive deficits [**6**]. 

The repeated failures, the behavioral changes keep them from readapting to their social and familial environment [**32**]. However, we should say that familial support and cooperation with medical staff would facilitate the patient’s reinsertion.

Irritability is a major cause for deficits in social and professional reintegration and in daily routine in patients with MHI. This irritability is associated with cortical lesions, but no lateralization or localization [**27**]. In our study, are identified these cortical lesions through psychological tests (WISC subtest "image completion" and "object assembling").

In children, because of the global functioning of the brain, psychical functions and processes are altered by the trauma even through microscopically lesions. The absence of abnormalities' images is not equivalent with the absence of abnormality because micro-structures can support the damages reflected as diaskisis's effects and expressed in any kind of behavior deficits.

Reintegration difficulties, tolerated by the patient after MHI, are generated by fear, affective lability, low stress tolerance and increased anxiety. These are the factors that predispose to temperamental and behavioral changes, which will gradually decrease the subject’s quality of life and will have repercussions on their families. Psychotherapeutically intervention is recommended (here are indicated and strategies of family system therapy) [**22**]. Psychotherapy is an effective intervention starting in the patient's hospitalization.

This intervention must to be included in the neurosurgeon's investigation protocol as a unique counseling, in order to inform the patient and his family regarding his evolution or as a psychotherapy program adapted to each patient, based on some individual investigations. In our study, the patients benefited of these services as a free offer and we obtained good results in 74% between children with falls.

## Conclusions

1. The more important affected were psychological functions and cognitive processes at lower age because the brain is functioning global.

2. The dependent structure is a predisposition for MHI in children.

3. For a good evolution, the attitude of parents and familial conditions are very important because, if disturbances exist, children develop behavior changes with secondary benefits.

4. For children who are developing in good conditions with specialized appropriate support and education, the symptoms will be disappearing sooner than 1 year.
